# PERSISTENT ORGANIC POLLUTANTS: Melting Glaciers Release Frozen Toxicants

**DOI:** 10.1289/ehp.117-a538

**Published:** 2009-12

**Authors:** Bob Weinhold

**Affiliations:** **Bob Weinhold**, MA, has covered environmental health issues for numerous outlets since 1996. He is a member of the Society of Environmental Journalists

The melting of glacial ice that formed in the middle of the twentieth century may be a source of a cocktail of persistent, bioaccumulative toxic substances that can threaten human health and the environment, according to a study by Christian Bogdal, a postdoctoral research fellow at the Swiss Federal Institute of Technology, Zürich, and colleagues in the 1 November 2009 issue of *Environmental Science & Technolog*y. Their findings, along with those of a handful of other studies, suggest the release of toxics once bound within glaciers may be a little-recognized consequence of ongoing climate change.

The Swiss team analyzed sediment samples taken from Lake Oberaar, a glacier-fed lake in central Switzerland. They measured sediment levels of several pollutants that would have entered the lake over the period 1953–2006 and compared the measurements with those from three lower-altitude Swiss lakes that aren’t glacier fed. The substances analyzed included 17 dioxins and furans, 18 polychlorinated biphenyls (PCBs), 10 synthetic musk compounds, DDT and two of its transformation products (DDE and DDD), 4 additional organochlorine pesticides (hexachlorobenzene, hexachlorocyclohexane, dieldrin, and heptachlor epoxide) and their transformation products, and polychlorinated naphthalenes.

The Lake Oberaar and lower-altitude samples reflected a generally consistent pattern of increased influx of the compounds into the lakes from the 1950s through a peak in the 1960s–1970s, followed by a decrease to relatively low levels in the 1980s and 1990s. The exception was musk compounds, whose sediment influx was fairly steady from the 1950s to the mid-1990s. These patterns parallel the widespread increase in production and use of these substances from the 1950s through the 1970s, and the subsequent decline (except in the case of musk compounds) as concerns about toxic effects often resulted in restrictions or bans on use. These patterns also mesh with the premise that the primary source of the sediment toxics to that point was deposition of airborne pollutants generated in urban, industrial, and agricultural areas.

Beginning in the late 1990s, however, the influx of all the compounds into Lake Oberaar—but not the lower-altitude lakes—increased moderately to sharply. In some cases, the new peak influxes were 2–5 times higher than the 1960s–1970s peaks. These increases coincide with a total reduction in Alpine glacier volume of about 12% between 1999 and 2008, according to a report by Daniel Farinotti and colleagues in the August 2009 issue of *Global and Planetary Change*. About one-quarter of that reduction occurred following the unusually hot summer of 2003.

Bogdal and colleagues hypothesize that the sources of the later contaminant influxes they observed likely were not distant, because production and use of these chemicals had decreased substantially. Instead, they conclude the glacial meltwater was the source. Studies they are conducting at Lake Oberaar and elsewhere support this hypothesis, says Bogdal.

The evidence to date suggests the release of these and other persistent toxics, such as lead and mercury, may be a concern for many glaciated settings. Among areas of potential concern, say Bogdal and other experts in this field, are locations in the Arctic region, Antarctica, the Alps, the Himalayas, and the Caucasus, Andes, Rocky, Cascade, and Sierra Nevada mountain ranges. “Melting of glaciers is releasing a huge amount of water containing dangerous contaminants used in the past,” says Roberta Bettinetti, an assistant professor of freshwater ecology at Italy’s University of Insubria. “Now these pollutants can contaminate great basins even at low altitudes where water is used for drinking and fishing purposes.”

In the October 2008 issue of *Chemosphere*, Bettinetti and colleagues reported on their study of the toxic effects of melting glaciers on biota of two southern Alpine lakes. They found that the amount of DDT and its metabolites released by melting glacial ice increased the concentrations in lake mussels and fish above the threshold considered safe for human consumption. Silvana Galassi, a professor of ecology at the University of Milan and coauthor of that report, recommends implementation of monitoring to identify areas where mitigation, such as limiting fish consumption or avoiding disturbance of sediments, may be warranted.

## Figures and Tables

**Figure:**
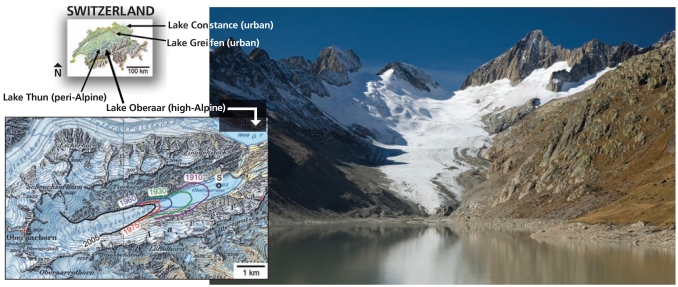
**The retreat of the Oberaar Glacier is not a new phenomenon, but Bogdal and colleagues point out that ice formed in periods of higher pollution can result in “important” releases of contaminants upon melting.** Figure, left: Bogdal C et al. 2009. Environ Sci Technol 43(21):8173–8177.

